# Supplementary material for: Losing ground in the field: An exploratory analysis of the relationship between work and mental health amongst women in conflict affected Democratic Republic of the Congo
Source: PLoS One. 2023 Apr 21;18(4):e0284088. doi: 10.1371/journal.pone.0284088 (PMC10121043; doi:10.1371/journal.pone.0284088)
Supplement: S1 Table — (DOCX) [file pone.0284088.s001.docx]

Table S1: Balance tests between the therapy and no-therapy groups at baseline

|  |  | (1) |  | (2) |  | (3) | t-test |
| --- | --- | --- | --- | --- | --- | --- | --- |
|  |  | No-therapy |  | Therapy |  | Total | Difference |
| Variable | N | Mean/SD | N | Mean/SD | N | Mean/SD | (1)-(2) |
| PTSD checklist score | 528 | 1.953 | 525 | 1.964 | 1053 | 1.959 | -0.011 |
|  |  | [0.565] |  | [0.596] |  | [0.581] |  |
| Probable PTSD | 528 | 0.701 | 525 | 0.703 | 1053 | 0.702 | -0.002 |
|  |  | [0.458] |  | [0.457] |  | [0.458] |  |
| HSCL-25 score for combined depression and anxiety | 528 | 2.138 | 525 | 2.141 | 1053 | 2.140 | -0.003 |
|  |  | [0.541] |  | [0.549] |  | [0.545] |  |
| Probable depression or anxiety | 528 | 0.794 | 525 | 0.792 | 1053 | 0.793 | 0.001 |
|  |  | [0.405] |  | [0.406] |  | [0.405] |  |
| Local functioning impairment index | 528 | 1.436 | 525 | 1.377 | 1053 | 1.407 | 0.060 |
|  |  | [0.691] |  | [0.682] |  | [0.687] |  |
| PTSD checklist score - experiencing | 528 | 2.110 | 525 | 2.115 | 1053 | 2.113 | -0.005 |
|  |  | [0.712] |  | [0.745] |  | [0.728] |  |
| PTSD checklist score - avoidance | 528 | 1.862 | 525 | 1.861 | 1053 | 1.861 | 0.001 |
|  |  | [0.606] |  | [0.616] |  | [0.611] |  |
| PTSD checklist score - arousal | 528 | 1.956 | 525 | 1.989 | 1053 | 1.973 | -0.033 |
|  |  | [0.666] |  | [0.710] |  | [0.689] |  |
| Worked in the last 7 days | 528 | 0.881 | 525 | 0.867 | 1053 | 0.874 | 0.014 |
|  |  | [0.324] |  | [0.340] |  | [0.332] |  |
| Paid work last 7d (=1 if worked with earnings; =0 if worked w/o earnings) | 465 | 0.806 | 455 | 0.756 | 920 | 0.782 | 0.050* |
|  |  | [0.396] |  | [0.430] |  | [0.413] |  |
| Paid work last 7d (=1 if worked with earnings; =0 if not worked) | 438 | 0.856 | 414 | 0.831 | 852 | 0.844 | 0.025 |
|  |  | [0.351] |  | [0.375] |  | [0.363] |  |
| Unpaid work last 7d (=1 if worked with no earnings; =0 if not worked) | 152 | 0.586 | 179 | 0.609 | 331 | 0.598 | -0.023 |
|  |  | [0.494] |  | [0.489] |  | [0.491] |  |
| Respondent has a secondary activity | 493 | 0.479 | 487 | 0.466 | 980 | 0.472 | 0.013 |
|  |  | [0.500] |  | [0.499] |  | [0.499] |  |
| Total earnings per hour in the last 7 days (winsorized 1%) | 447 | 0.127 | 429 | 0.132 | 876 | 0.129 | -0.005 |
|  |  | [0.149] |  | [0.176] |  | [0.163] |  |
| Total earnings generated in the last 7d (winsorized 1%) | 493 | 3.444 | 485 | 3.641 | 978 | 3.542 | -0.197 |
|  |  | [4.388] |  | [5.427] |  | [4.929] |  |
| Total hours worked in the last 7 days | 465 | 32.110 | 455 | 32.358 | 920 | 32.233 | -0.249 |
|  |  | [16.679] |  | [17.724] |  | [17.195] |  |
| Self-employed in the main activity | 504 | 0.508 | 495 | 0.489 | 999 | 0.498 | 0.019 |
|  |  | [0.500] |  | [0.500] |  | [0.500] |  |
| Wage-worker status in the main activity | 504 | 0.393 | 495 | 0.382 | 999 | 0.387 | 0.011 |
|  |  | [0.489] |  | [0.486] |  | [0.487] |  |
| Farming is the main activity | 504 | 0.766 | 495 | 0.756 | 999 | 0.761 | 0.010 |
|  |  | [0.424] |  | [0.430] |  | [0.427] |  |
| On-farm and self-employed in the main activity | 504 | 0.375 | 495 | 0.339 | 999 | 0.357 | 0.036 |
|  |  | [0.485] |  | [0.474] |  | [0.479] |  |
| Off-farm and self-employed in the main activity | 504 | 0.133 | 495 | 0.149 | 999 | 0.141 | -0.017 |
|  |  | [0.340] |  | [0.357] |  | [0.348] |  |
| On-farm and wage-worker status in the main activity | 504 | 0.296 | 495 | 0.293 | 999 | 0.294 | 0.003 |
|  |  | [0.457] |  | [0.456] |  | [0.456] |  |
| Off-farm and wage-worker status in the main activity | 504 | 0.097 | 495 | 0.089 | 999 | 0.093 | 0.008 |
|  |  | [0.297] |  | [0.285] |  | [0.291] |  |

Note: Table shows adjusted means, controlling for: strata, wealth index, livestock ownership index, age, household size, sex of household head, level of education, marital status. The value displayed for t-tests are the differences in the means across the groups. ***, **, and * indicate significance at the 1, 5, and 10 percent critical level.
