# Supplementary material for: Losing ground in the field: An exploratory analysis of the relationship between work and mental health amongst women in conflict affected Democratic Republic of the Congo
Source: PLoS One. 2023 Apr 21;18(4):e0284088. doi: 10.1371/journal.pone.0284088 (PMC10121043; doi:10.1371/journal.pone.0284088)
Supplement: Attachment — Submitted filename: Revision plos article .docx [file pone.0284088.s006.docx]

-The manuscript was well written and the subject is very interesting. With the context of the Democratic Republic of Congo, these data will help improve the care of women who have suffered violence.

**Introduction**

- The introduction is very long. We must focus on the elements that will justify the realization of this work.

-From line 113 to 121, you mention the purpose of this study, but after that comes a paragraph on the context of the study. It is better to first talk about the context before detailing the goal because it also takes into account the context of the study. The goal may be different depending on whether you work in one environment or another.

-On line 129, you cite a 2010 study without specifying the target study population.

-For a logical sequence of ideas and to respect the chronology, it would be good to move the sentence up, from line 138 to line 141, to line 136, just before starting the quote from the 2018 study.

**Methods**

-On line 156: correct the word “nartional” by removing the “r” between “a” and “t”.

-At this point, it is necessary to specify which ethics committee it is: that of the school of public health or the ministry of health.

-Better specify how the diagnosis of PTSD was made.

- A brief description of the different sites where the study was carried out is missing.

-Line 221: the "USD" must be written in full during the first quote and then abbreviated.

- In the last paragraph of the methods, detail, if this has been done, the different therapeutic modalities from which the respondents would have benefited.

**Results**

-Line 288: modify the title: instead of saying "descriptive statistics", say rather "socio-epidemiological data".

-Tables 7 and 8: add to the legend the meaning of Y.

**Discussion**

-Before talking about the limitations of the study, insert a paragraph to propose your model for monitoring women who have suffered violence.
